# Exploration of Diverse Secondary Metabolites From *Streptomyces* sp. YINM00001, Using Genome Mining and One Strain Many Compounds Approach

**DOI:** 10.3389/fmicb.2022.831174

**Published:** 2022-02-10

**Authors:** Tao Liu, Zhen Ren, Wei-Xun Chunyu, Gui-Ding Li, Xiu Chen, Zhou-Tian-Le Zhang, Hui-Bing Sun, Mei Wang, Tian-Peng Xie, Meng Wang, Jing-Yuan Chen, Hao Zhou, Zhong-Tao Ding, Min Yin

**Affiliations:** ^1^School of Medicine, School of Chemical Science and Technology, Yunnan University, Kunming, China; ^2^School of Agriculture and Life Sciences, Kunming University, Kunming, China; ^3^Department of Pathogen Biology and Immunology, Kunming Medical University, Kunming, China; ^4^College of Pharmacy, Dali University, Dali, China

**Keywords:** *Peperomia dindygulensis*, endophytic actinomycetes, genome mining, OSMAC approach, secondary metabolites

## Abstract

A talented endophytic bacteria strain YINM00001, which showed strong antimicrobial activity and multiple antibiotic resistances, was isolated from a Chinese medicinal herb *Peperomia dindygulensis* Miq. Phylogenetic analysis based on 16S rRNA gene sequences demonstrated that strain was closely related to *Streptomyces anulatus* NRRL B-2000T (99.93%). The complete genome of strain YINM00001 was sequenced. The RAxML phylogenomic tree also revealed that strain YINM00001 was steadily clustered on a branch with strain *Streptomyces anulatus* NRRL B-2000T under the 100 bootstrap values. The complete genome of strain YINM00001 consists of an 8,372,992 bp linear chromosome (71.72 mol% GC content) and a 317,781 bp circular plasmid (69.14 mol% GC content). Genome mining and OSMAC approach were carried out to investigate the biosynthetic potential of producing secondary metabolites. Fifty-two putative biosynthetic gene clusters of secondary metabolites were found, including the putative cycloheximide, dinactin, warkmycin, and anthracimycin biosynthetic gene clusters which consist with the strong antifungal and antibacterial activities exhibited by strain YINM00001. Two new compounds, peperodione (**1**) and peperophthalene (**2**), and 17 known compounds were isolated from different fermentation broth. Large amounts and high diversity of antimicrobial and/or anticancer compounds cycloheximide, dinactin, anthracimycin, and their analogs had been found as predicted before, which highlights strain YINM00001 as an ideal candidate for further biosynthetic studies and production improvement of these valuable compounds. Meanwhile, several gene clusters that were highly conserved in several sequenced actinomycetes but significantly different from known gene clusters might be silent under proceeding fermentation conditions. Further studies, such as heterologous expression and genetic modification, are needed to explore more novel compounds from this talented endophytic *Streptomyces* strain.

## Introduction

The development of new drugs lags far behind the outbreak of infectious diseases and cancers. It is urgent to find novel natural products. Plenty of bioactive natural products were found from actinomycetes, including clinical and agricultural antibiotics, antitumor drugs, immunosuppressants, and other active drugs. Actinomycetes are responsible for about 10,100 of the 22,500 bioactive microbial metabolites that have been discovered so far; about 150 compounds were directly applied to humans, animals, and agriculture (Berdy, 2005; Newman and Cragg, 2020). However, with the accumulation of strains and compounds year by year, the probability of rediscovery of studied strains and known compounds is greatly increased. In addition, time-consuming and guideless traditional fermentation makes it difficult to find novel natural products. Using new research methods to explore new actinomycetes trains isolated from new habitats might be a new way to discover novel natural products (Shen, 2015; Newman and Cragg, 2020).

Endophytic bacteria, which live in almost all studied plants, are important components of the host ecosystem. Many endophytes could give beneficial feedback to their host in different ways, including growth promotion, pathogen suppression, contaminants remove, phosphate solubilization, nitrogen fixation, etc. (Assmus et al., 1995; Sessitsch et al., 2002; Hardoim et al., 2008). During the long time interaction with their host, Chinese medicinal herb derived endophytic bacteria, especially actinomycetes strains, could produce various bioactive natural products that make them valuable resources for drug leads discovery.

Chinese medicinal herbs have been used as anti-infection and anti-cancer drugs for thousands of years. Many natural products isolated from Chinese medicinal herbs showed considerable antibacterial, antifungal, antiviral, and antitumor activities (Lee, 2000). *Peperomia dindygulensis* Miq. is a fleshy herb mainly found in southern China. Compounds with antitumor, anti-inflammatory, and antiviral activities had been isolated from *Peperomia dindygulensis*, including Secolignans, tetrahydrofuran lignans, chromones, and acylcyclohexane-1,3-diones. It is commonly used for treatment of cough, asthma, measles, burns, and cancers in southwest China (Wang et al., 2012). However, the endophytic bacteria of *Peperomia dindygulensis* are still poorly characterized.

On the basis of the deepening understanding of the biosynthesis mechanism of some secondary metabolites, genome mining has become a new way to explore new natural products. By applying the knowledge of biosynthetic pathways, potential gene clusters can be mined from the genome, skeletal structure of the relative products can be predicted, and some gene clusters can be activated in a variety of ways, so as to discover new natural products. Meanwhile, because some bacteria can potentially produce many more metabolites than they do under determined conditions, the “one strain many compounds” (OSMAC) approach had been widely used in recent years (Bode et al., 2002; Romano et al., 2018). Therefore, using genomic mining and OSMAC approach together to investigate new actinomycetes strains from new habitats may get better results.

In this study, endophytic bacteria, including *Streptomyces* strains, were isolated from *Peperomia dindygulensis* Miq. One of the *Streptomyces* strains, designed YINM00001, showed strong antimicrobial activity and multi-antibiotics resistance and was chosen for secondary metabolites discovery by using genome mining and OSMAC approach. Totally, 19 natural products including two new compounds were found from different fermentation broths of strain YINM00001. Some compounds had been predicted by genome mining before isolation, and several active compounds were consistent with the activities exhibited by strain YINM00001. Our study showed the great potential of genome mining and OSMAC in natural products exploration from novel strains.

## Materials and Methods

### Bacterial Isolation

Fresh *Peperomia dindygulensis* were collected from the Chinese medicinal herb garden in Kunming University, Yunnan Province, China. Ten healthy plants, including leaves, stems, and roots, were randomly chosen from the garden. All the samples were stored at 4^°^C and processed within 12 h. The whole plants were washed with tap water and distilled water several times, and then immersed in 70% ethanol (v/v) and sodium hypochlorite solution (0.5% available chlorine) for 1 min, respectively. After being washed with distilled water thoroughly three times, the whole plants were smashed into small pieces with a sterilized grinder (Ren et al., 2018). The juice and residues were plated onto ISP2 agar (soytone 4 g, malt extract 10 g, glucose 4 g, agar 20 g, H_2_O 1000 ml, pH = 7.2) and Gause’s synthetic agar (soluble starch 20 g, sodium chloride 0.5 g, ferrous sulfate 0.01 g, potassium nitrate 1 g, sipotassium hydrogen phosphate 0.5 g, magnesium sulfate 0.5 g, agar 15 g, pH = 7.2) supplemented with nystatin and nalidixic acid (50 μg/ml each) to isolate actinomycetes strains. Then, the plates were incubated at 28^°^C for up to a month. The actinomycete strains were picked out and purified on ISP2 plates with nystatin and nalidixic acid. The purified cultures were preserved in ISP2 slants and 20% (v/v) glycerol tubes and stored at 4 and −80^°^C, respectively, for further using. Genomic DNA isolation, 16S rRNA gene amplification, and sequencing were done as described before (Li et al., 2021).

### Antimicrobial Activity and Antibiotic Resistance Assay

Fresh cultures of isolated strains were inoculated on ISP2 agar disks and incubated at 28^°^C for a week. The liquid cultures of activated pathogens, including *Escherichia coli* (CGMCC 1.2385), *Pseudomonas aeruginosa* (CGMCC 1.2387), *Bacillus subtilis* (CGMCC 1.1849), *Staphylococcus aureus* (CGMCC 1.2386), *Mycobacterium tuberculosis* (ATCC 25177), *Fusarium oxysporum* (MW149127.1), *Candida albicans* (CGMCC 2.2086), and *Fusarium fulcrum* (MW149128.1), were mixed well with Mueller-Hinton Agar (beef extract powder 2 g, casein hydrolyzate 17.5 g, starch 1.5 g, agar 12 g, pH = 7.3) 1:1 v/v and then spread on the top of ISP2 agar. Once the top agar had solidified, about 5 mm diameter ISP2 agar with a single colony of each isolated strains was placed on the top agar and incubated at 28^°^C for a week.

Fresh cultures of isolated strains were inoculated on ISP2 agar supplemented with chloramphenicol (50 μg/ml), kanamycin (200 μg/ml), levofloxacin (50 μg/ml), vancomycin (50 μg/ml), rifampicin (50 μg/ml), oxytetracycline (50 μg/ml), apramycin (50 μg/ml), and bacitracin (50 μg/ml) and incubated at 28^°^C for 5 days.

### Genome Sequencing and Mining

A single colony of strain YINM00001 was inoculated into 50 ml Tryptic Soy Broth (TSB) medium (casein tryptone 17 g, soy peptone 5 g, sodium chloride 5 g, D-glucose 2.5 g, dipotassium phosphate 2.5 g, H_2_O up to 1,000 ml, pH = 7.3) for 36 h at 28^°^C with 200 rpm vigorous shaking. DNA isolation and purification in strain YINM00001 were carried out according to standard procedures (Pospiech and Neumann, 1995). The quality and quantity of purified genomic DNA were analyzed by using NanoDrop 2000 spectrophotometer (Thermo Scientific, Wilmington, DE, United States) and 0.8% agarose gel electrophoresis.

For Pacbio sequencing, genomic DNA was sheared to 8–10 kb length fragments randomly and a genomic DNA library was constructed. Pacbio clean data were generated by sequencing platform; all the reads were assembled and checked using HGAP software (Rhoads and Au, 2015). For Illumina Hiseq sequencing, genomic DNA was sheared to about 400 bp length fragments randomly and a genomic DNA library was constructed. PE150 pair-end sequencing was carried out, and clean data were obtained. SOAPdenovo V2.04 (Luo et al., 2012) was used to check and assemble all the reads.

The DNA G+C mol% value was obtained from the genomic sequence. The protein coding sequences of the chromosome and plasmid were predicted by Glimmer (v3.02) (Delcher et al., 2007) and GeneMarkS, respectively (Besemer et al., 2001). tRNA and rRNA were predicted with tRNAscan-SE v2.0 (Chan et al., 2021) and Barrnap,^1^ respectively. EzTaxon-e database (Yoon et al., 2017) was used to identify phylogenetic neighbors of isolated strains. Phylogenetic tree of 16S rRNA gene was reconstructed based on neighbor-joining (NJ) (Saitou and Nei, 1987) in mega software (version 7.0.14) (Kumar et al., 2016). Evolutionary distances for the analyses were calculated using the Kimura two-parameter model (Kimura, 1980). The genome tree based on the concatenated sequences of 557 protein marker genes was reconstructed using RAxML (Stamatakis, 2014), and fast bootstrapping (Stamatakis et al., 2008) was used to generate the support values in the tree.

Clusters of Orthologous Groups of proteins (COG) and Gene Ontology (GO) programs (Tatusov et al., 2003; Gene Ontology Consortium, 2021) were used to analyze the function of annotated genes. Kyoto Encyclopedia of Genes and Genomes (KEGG) PATHWAY analysis (Kanehisa and Goto, 2000) was carried out to determine the key potential pathways in strain YINM00001. CRISPR finder platform (Grissa et al., 2007) was used to identify CRISPR-Cas sequences on chromosome. PHAST (Zhou et al., 2011) was used to identify putative prophages on the chromosome. Tandem repeats finder (Benson, 1999) and Repeatmasker^2^ were used to identify simple tandem repeats and interspersed repeats, respectively. Genomic islands were predicted with IslandViewer 4 (Bertelli et al., 2017). Transposable elements were identified with ISEScan (Xie and Tang, 2017). ResFinder (Zankari et al., 2012) and the Comprehensive Antibiotic Resistance Database (CARD) (Jia et al., 2017) were used to predict resistance genes. The VFDB database (Chen et al., 2016) was used to predict bacterial virulence factors.

The biosynthetic gene clusters for putative secondary metabolites were identified using the antiSMASH 5.0 (Blin et al., 2019) and PRISM 4 programs (Skinnider et al., 2020) and then verified by manual inspection.

### One Strain Many Compounds Strategy Fermentation

For small scale fermentation, the strain YINM00001 was activated in MS medium (soya flour 20 g, mannitol 20 g, agar 20 g, H_2_O up to 1,000 ml, pH = 7.0) at 28^°^C for 4 days. The activated strain was inoculated into 500 ml Erlenmeyer flasks containing 100 ml of TSB medium and cultured for 4 days at 28^°^C and 200 rpm. Then, 5.0 ml of the seed culture was transferred into 500 ml Erlenmeyer flasks containing 100 ml of 10 different types of fermentation media (1#–10#, data not show), respectively.

For large scale fermentation, seed cultures of strain YINM00001 were prepared as described above. Then, 5.0 ml of the seed cultures were transferred into 500 ml Erlenmeyer flasks containing 100 ml of 4# and 9# fermentation media (4# medium, soluble starch 40 g, corn extract 10 g, monopotassium phosphate 0.5 g, magnesium sulfate 0.25 g, zinc sulfate heptahydrate 40 mg, methionine 0.1 g, vitamin B12 0.1 g, calcium carbonate 5 g, H_2_O up to 1000 ml, pH = 7.2; 9# medium, glucose 10 g, glycerol 10 ml, corn extract 2.5 g, peptone 5 g, soluble starch 10 g, yeast extract 2 g, calcium carbonate 3 g, sodium chloride 1 g, H_2_O up to 1,000 ml, pH = 7.3), respectively, and cultured for 10 days at 28°C and 200 rpm (15 L each).

### Isolation and Identification of Compounds

The broth was extracted with equal volume ethyl acetate (EtOAc) three times, and the solvent was removed under vacuum to obtain the EtOAc extract. The crude extract of 4# medium fermentation broth (10.2 g) was separated into four fractions (Fr.1–Fr.4) by LiChroprep RP-18 column, eluting stepwise with a MeOH-H_2_O gradient (30% MeOH, 50% MeOH, 70% MeOH, and 100% MeOH). Fr.1 was fractionated by a Sephadex LH-20 column with MeOH to afford Fr.1-1 to Fr.1-4. Then, Fr.1-1 was separated by a Sephadex LH-20 column with MeOH to obtain **10** (34.2 mg). Fr.1-3 was chromatographed using a silica gel column (petroleum ether-acetone, 150:1 to 20:1) to obtain **17** (20.5 mg) and **18** (26.3 mg). Fr.2 was separated by a Sephadex LH-20 column with MeOH to give **3** (36.8 mg) and **4** (15.9 mg). Fr.3 was divided into three parts (Fr.3-1 to Fr.3-3) by a Sephadex LH-20 column with MeOH. Fr.3-1 was purified by a silica gel column with CH_2_Cl_2_-EtOAc (80:1-9:1) to afford **15** (13.5 mg) and **16** (15.8 mg). Fr.3-2 was separated by a silica gel column (petroleum ether-acetone, 50:1 to 1:1) to obtain **1** (8.9 mg). Fr.3-3 was chromatographed using a silica gel column (CH_2_Cl_2_-MeOH, 100:1-10:1) to give **5** (8.5 mg) and **6** (8.5 mg). Fr.4 was divided into three parts (Fr.4-1 to Fr.4-3) by a Sephadex LH-20 column with MeOH. Fr.4-1 was fractionated by a silica gel column with CH_2_Cl_2_-MeOH (100:1-10:1) to afford **19** (16.5 mg). Fr.4-2 was separated by a Sephadex LH-20 column with MeOH to give **11/12** (20.6 mg) and **13/14** (17.9 mg). Fr.4-3 was chromatographed using a silica gel column (CH_2_Cl_2_-MeOH, 130:1-10:1) to yield **7** (9.5 mg), **8** (14.3 mg), and **9** (11.9 mg). The crude extract of 9# medium fermentation broth (9.1 g) was also separated into four fractions (Fr.1–Fr.4) by LiChroprep RP-18 column, eluting stepwise with a MeOH-H_2_O gradient (30% MeOH, 50% MeOH, 70% MeOH, and 100% MeOH). Fr.3 was divided into two parts (Fr.3-1 to Fr.3-2) by a Sephadex LH-20 column with MeOH. Fr.3-2 was chromatographed using a silica gel column (CH_2_Cl_2_-MeOH, 100:1-20:1) to give **2** (4.2 mg).

The 1D and 2D NMR spectra were measured on a Bruker Avance-400 MHz instrument (Bruker, Karlsruhe, Germany) with tetramethylsilane as the internal standard. HRESIMS data were obtained by an Agilent 1200 Q-TOF mass instrument (Agilent, Santa Clara, CA, United States). Silica gel (200-300 mesh, Qingdao Marine Chemical Group Co., Qingdao, China), Lichroprep RP-18 gel (40-63 mm, Merck, Darmstadt, Germany), and Sephadex LH-20 (GE Healthcare Bio-Science AB, Uppsala, Sweden) were used for column chromatography (CC). Thin-layer chromatography (TLC) was performed on silica gel GF254 plates (Qingdao Haiyang Chemical Co., Ltd., Qingdao, China) and visualized by spraying with anisaldehyde-H_2_SO_4_ reagent. All solvents used for CC were of analytical grade from Chengdu Titan Chron Chemical Co., Ltd. (Chengdu, China).

## Results and Discussion

### Strain Isolation and 16S rRNA Gene Sequence

A total of 39 endophytic bacteria, including 12 actinomycetes, were isolated from *Peperomia dindygulensis*. Phylogenetic analyses of the 16S rRNA gene sequences showed that strains *Streptomyces* sp. YM9, *Streptomyces* sp. YM74, *Streptomyces* sp. YM75, *Streptomyces* sp. YM77, *Streptomyces* sp. YM79, *Streptomyces* sp. YM80, *Streptomyces* sp. YM81, *Streptomyces* sp. YM83, *Streptomyces* sp. YM84, *Streptomyces* sp. YM85, *Streptomyces* sp. YM86, and *Streptomyces* sp. YINM00001 were closely related to *Streptomyces setonii*, *Streptomyces hydrogenans*, *Streptomyces coelescens*, *Streptomyces aurantiacus*, *Streptomyces bottropensis*, *Streptomyces cellulosae*, *Streptomyces lusitanus*, *Streptomyces albogriseolus*, *Streptomyces hydrogenans*, *Streptomyces speibonae*, *Streptomyces aureus*, and *Streptomyces fulvissimus*, respectively. Probably due to the thin stems, roots, and leaves of *Peperomia dindygulensis*, a large number of vulnerable bacteria were killed by surface sterilization with ethanol and sodium hypochlorite solution. Only a few dozen bacteria were isolated. *Streptomyces* spores are highly resistant to sterilization, and isolation media ISP2 and Gause’s synthetic agar are suitable for the growth of actinomycetes, which might lead to separation of a variety of *Streptomyces* strains.

### Antimicrobial Activity and Antibiotic Resistance of *Streptomyces* YINM00001

Strains of *Streptomyces* genius could produce various types of antibiotics. In this study, eight pathogens, including *Escherichia coli* (CGMCC 1.2385), *Pseudomonas aeruginosa* (CGMCC 1.2387), *Bacillus subtilis* (CGMCC 1.1849), *Staphylococcus aureus* (CGMCC 1.2386), *Mycobacterium tuberculosis* (ATCC 25177), *Fusarium oxysporum* (MW149127.1), *Candida albicans* (CGMCC 2.2086), and *Fusarium fulcrum* (MW149128.1), were chosen for antimicrobial activity screening. Among them, *Streptomyces* sp. YINM00001 showed strong inhibition activities against several gram positive bacterial and fungal pathogens, including *S. aureus*, *M. tuberculosis*, *F. oxysporum*, and *C. albicans*. Other strains showed certain antimicrobial activity against a few pathogens (Table 1). Antimicrobial activity test results indicated that *Streptomyces* sp. YINM00001 might have the capacity to produce different types of active antibiotics.

**TABLE 1:** Antimicrobial activity assay of Streptomyces strains.

	Pathogens							
	*Escherichia coli* (CGMCC 1.2385)	*Pseudomonas aeruginosa* (CGMCC 1.2387)	*Bacillus subtilis* (CGMCC 1.1849)	*Staphylococcus aureus* (CGMCC 1.2386)	*Mycobacterium tuberculosis* (ATCC 25177)	*Fusarium oxysporum* (MW149127.1)	*Candida albicans* (CGMCC 2.2086)	*Fusarium fulcrum* (MW149128.1)
**Isolates**								
*Streptomyces* sp. YM83	–	–	++	–	–	–	–	–
*Streptomyces* sp. YM77	+	–	–	–	–	–	–	–
*Streptomyces* sp. YM86	+	–	–	+	–	–	–	–
*Streptomyces* sp. YM79	–	–	–	++	–	–	–	–
*Streptomyces* sp. YM75	–	+–	–	+	–	–	–	–
*Streptomyces* sp. YINM00001	–	–	–	+++	++	+++	+++	–
*Streptomyces* sp. YM74	+	–	–	–	–	–	–	–
*Streptomyces* sp. YM84	–	–	++	–	–	–	–	–
*Streptomyces* sp. YM81	+	–	–	+	–	–	–	–
*Streptomyces* sp. YM9	–	–	–	–	–	–	–	–
*Streptomyces* sp. YM85	+	+	–	–	–	–	–	–
*Streptomyces* sp. YM80	+	–	–	–	–	–	–	–

*Diameter of inhibitory zone: +, <5 mm; ++, 5–10 mm; +++, >10 mm; -, no inhibitory zone.*

To avoid being killed by the antibiotics produced by the strains themselves, antibiotics resistant genes are often found within or beside the biosynthetic gene clusters. Testing the antibiotic resistance of the strains could unveil their possibility to produce antibiotics. Strain YINM00001 could grow well on ISP2 media with chloramphenicol, vancomycin, oxytetracycline, and bacitracin. Some of the remaining strains could grow on ISP2 media with different antibiotics (Table 2). Antibiotic resistance tests indicated that strain YINM00001 might produce chloramphenicol, glycopeptide, tetracycline, and polypeptide antibiotics. Based on the results of antimicrobial activity and antibiotic resistance screening, *Streptomyces* sp. YINM00001 was chosen for genome sequencing.

**TABLE 2:** Antibiotic resistance assay of Streptomyces strains.

	Antibiotics							
	Chloramphenicol (50 μg/ml)	Kanamycin (200 μg/ml)	Levofloxacin (50 μg/ml)	Vancomycin (50 μg/ml)	Rifampicin (50 μg/ml)	Oxytetracycline (50 μg/ml)	Apramycin (50 μg/ml)	Bacitracin (50 μg/ml)
**Isolates**								
*Streptomyces* sp. YM83	–	–	–	–	–	–	+	++
*Streptomyces* sp. YM77	–	–	–	–	+	–	+	+
*Streptomyces* sp. YM86	–	–	–	–	+	+	–	–
*Streptomyces* sp. YM79	–	–	–	+	–	+	–	+
*Streptomyces* sp. YM75	–	–	–	–	–	–	–	–
*Streptomyces* sp. YINM00001	++	–	–	++	–	++	–	++
*Streptomyces* sp. YM74	–	–	–	+	–	–	–	++
*Streptomyces* sp. YM84	–	–	–	–	–	–	–	+
*Streptomyces* sp. YM81	–	–	–	–	–	–	–	–
*Streptomyces* sp. YM9	+	–	–	+	–	–	–	+
*Streptomyces* sp. YM85	–	–	–	+	+	–	–	–
*Streptomyces* sp. YM80	–	–	–	–	–	–	–	–

*Growth state: +, grow poorly; ++, grow well; -, did not grow.*

### Genome Sequencing and Analysis of *Streptomyces* sp. YINM00001

The complete genome of strain YINM00001 was obtained by using Pacbio RSII and Illumina Hiseq platform. Approximately 1.62 Gb Pacbio and 1.08 Gb Illumina Hiseq clean data were generated. The average depth of genome coverage was 187-fold. The complete genome of strain YINM00001 was composed of a linear chromosome of 8,372,992 bp with a GC content of 71.72 mol% (accession no. CP086102) and a circular plasmid of 317,781 bp with a GC content of 69.14 mol% (accession no. CP086103).

**FIGURE 1:**
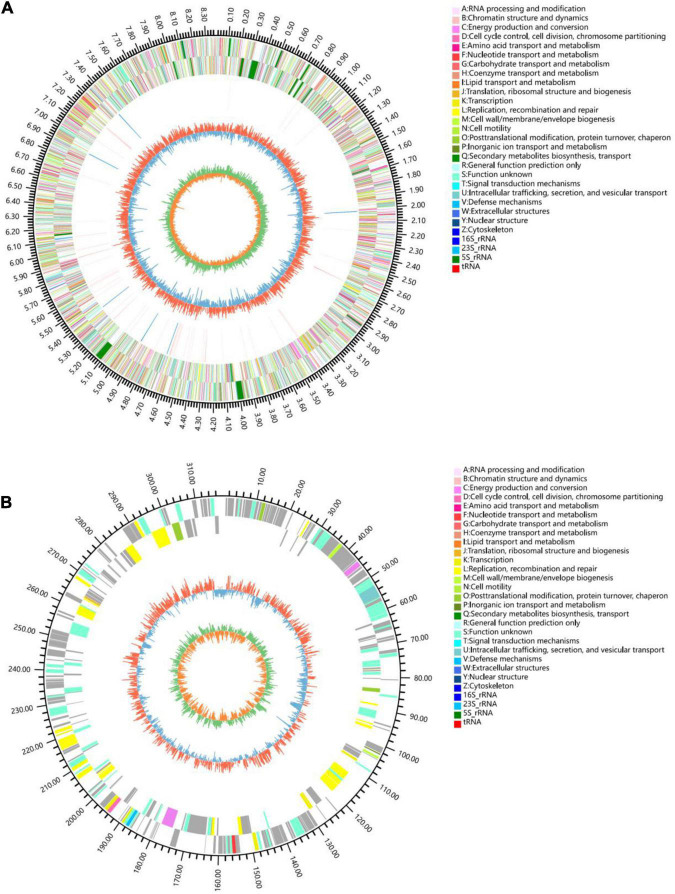
Genome map of strain YINM00001. **(A)** Chromosome. **(B)** Plasmid. Tracks (from outer to inner): (1) genome size, (2) forward strand gene, colored according to COG classification, (3) reverse strand gene, colored according to COG classification, (4) rRNA and tRNA, (5) GC content, (6) GC skew.

**FIGURE 2:**
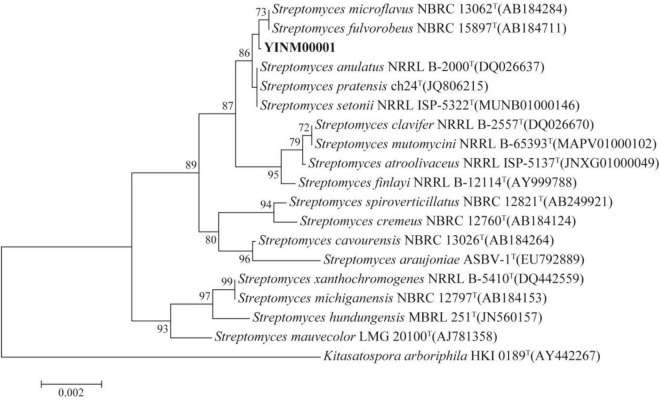
The neighbor-joining phylogenetic tree of strain YINM00001 and its closest relatives from the genus *Streptomyces* based on 16S rRNA genes. Bootstrap values (>70%) based on 1,000 resamplings are given at the nodes. *Kitasatospora arboriphila* NRRL HKI 0189T (accession no. AY442267) was used as outgroup. Bar, 0.002 substitutions per nucleotide position.

**FIGURE 3:**
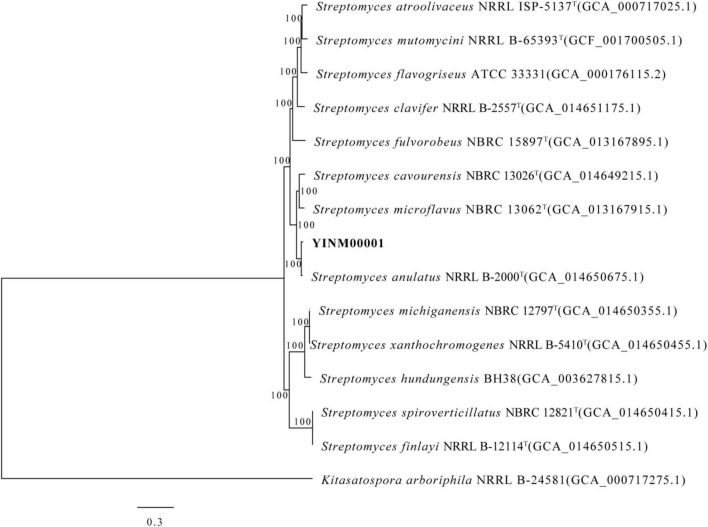
The RAxML phylogenomic tree of strain YINM00001 and its closest relatives from the genus *Streptomyces* based on marker genes. Bootstrap values (>70%) based on 1,000 resamplings are given at the nodes. *Kitasatospora arboriphila* NRRL B-24581 (accession no. GCA_000717275) was used as outgroup. Bar, 0.3 substitutions per nucleotide position.

The chromosome contained 7,506 predicted genes, including 18 rRNA genes, 67 tRNA genes, and 52 sRNA genes (Figure 1 and Table 3). Six 16S rRNA genes were identified in the chromosome of strain YINM00001. The sequence of the 16S rRNA gene was used for phylogenetic analysis. Phylogenetic analyses of the 16S rRNA gene sequences showed that strain YINM00001 was a member of the genus *Streptomyces*. It was evident from the NJ phylogenetic tree that strain YINM00001 formed a cluster with *Streptomyces fulvorobeus* NBRC 15897T and *Streptomyces microflavus* NBRC 13062T. It showed the highest 16S rRNA gene sequence similarity (99.93%) with *Streptomyces anulatus* NRRL B-2000T (Figure 2). The RAxML phylogenomic tree demonstrated that strain YINM00001 formed a cluster with strain *S. anulatus* NRRL B-2000T under the 100 bootstrap values (Figure 3).

**TABLE 3:** Genome features of strain YINM00001.

Feature	Chromosome characteristics
Chromosome size (bp)	8,372,992
GC content (%)	71.72%
Predicted genes	7,506
rRNA operons	18
tRNA genes	67
sRNA genes	52
Genes assigned to COG	5,628
Genes assigned to GO	5,165
Genes assigned to KEGG	2,714
CRISPR repeat regions	50
Prophages	2
Simple tandem repeats	1,031
Interspersed repeats	31
Genomic islands	20
Carbohydrate-active enzymes	242
Transposable element	1
Antibiotic resistance genes	446
Virulence factors	606
Secondary metabolite gene clusters	53
Plasmid size (bp)	317,781
Plasmid GC content (%)	69.14
Plasmid predicted genes	309

Among the identified genes, 5,628 and 5,165 genes were classified into functional categories based on clusters of orthologous genes of proteins (COG) and GO designation, respectively (Supplementary Figures 1, 2). A total of 2,714 genes of KEGG pathways were assigned (Supplementary Figure 3). Fifty putative CRISPR repeat regions were identified on the chromosome of strain YINM00001 (Table 3). Two incomplete prophase remnants containing 70 and 25 coding sequences, respectively, were detected on the chromosome; the length of prophages ranges from 8,264 bp to 28,169 bp (Table 3). In total, 1,031 simple tandem repeats and 31 interspersed repeats were assigned (Table 3). Twenty genomic islands containing 459 genes which may contribute to the diversification and adaptation of microorganisms were found (Table 3). A total of 242 genes were assigned to be carbohydrate-active enzymes, including 27 auxiliary activities, 5 carbohydrate-binding modules, 58 carbohydrate esterases, 91 glycoside hydrolases, 55 glycosyl transferases, and six polysaccharide lyases (Table 3). One transposable element was found, which contains a 1,568 bp transposase (Table 3). In total, 446 genes were identified as antibiotic resistance genes, including one chloramphenicol resistance gene, 21 glycopeptide resistance genes, 31 tetracycline resistance genes, and 15 bacitracin resistance genes, which was consistent with the antibiotics resistance test results (Table 3). A total of 606 putative virulence factors were found (Table 3).

The circular plasmid contained 309 predicted genes, including 83 function assigned genes and 226 hypothetical proteins (Figure 1).

### Genome Mining of Secondary Metabolites

Based on genome mining results, 52 putative biosynthetic gene clusters were found (Table 4), including 14 saccharides, 6 polyketides, 5 polyketide-non-ribosomal peptides, 5 terpenes, 4 non-ribosomal peptides, 4 fatty acids, 3 lantipeptides, 3 halogenated, 2 siderophores, 2 ectoines, 1 Ripps, 1 melanin, 1 butyrolactone, and 1 thiopeptide. Among them, 14 putative gene clusters showed high similarity (>70% of genes show similarity) to ectoine, dinactin, AmfS, melanin, cycloheximide, alkylresorcinol, SGR PTMs, geosmin, warkmycin, RP-1776, streptobactin, desferrioxamin B, and anthracimycin gene clusters. The presence of the putative cycloheximide (Yin et al., 2014), dinactin (Zhang et al., 2020), warkmycin (Helaly et al., 2013), and anthracimycin (Hensler et al., 2014) biosynthetic gene clusters was consistent with the strong antifungal and antibacterial activities of strain YINM00001. Three putative gene clusters showed moderate similarity (30–70% of genes show similarity) to hopene, nucleocidin, and acarviostatin gene clusters. The existence of these putative gene clusters indicated that strain YINM00001 offers the opportunity to produce these important antibiotics or their analogs. Twenty-three putative gene clusters showed low similarity (<30% of genes show similarity) to reported frigocyclinone, steffimycin D, formicamycins, C-1027, dechlorocuracomycin, stambomycin, collismycin A, enduracidin, phosphonoglycans, retimycin A, CDA, asukamycin, cyclothiazomycin, herboxidiene, coelimycin P1, kanamycin, herboxidiene, SF2575, caniferolide, ficellomycin, kanamycin, and calicheamicin gene clusters. The other 13 putative gene clusters were not conserved relative to any known cluster. Some putative gene clusters were highly conserved in several sequenced actinomycetes but were significantly different from known gene clusters. The existence of these cryptic secondary metabolite biosynthetic gene clusters implied that novel antibiotics might be found from strain YINM00001.

**TABLE 4:** Secondary metabolite clusters in strain YINM00001.

Cluster	Type	From	To	Most similar known biosynthetic gene cluster	Similarity
1	PKS-NRPS	84,576	136,796	Kanamycin	2%
2	PKS	193,333	260,857	Cycloheximide	100%
3	PKS	287,907	392,629	Alkylresorcinol	100%
4	Saccharide	396,814	437,537	Herboxidiene	9%
5	PKS-NRPS	479,532	531,991	Dutomycin	4%
6	PKS	550,475	596,892	C-1027	17%
7	PKS-NRPS	685,700	790,947	SGR PTMs	100%
8	NRPS	830,795	881,560	Nucleocidin	47%
9	Terpene	921,379	954,079	Hopene	69%
10	Fatty_acid	1,123,671	1,142,079	NA	
11	Fatty_acid	1,226,558	1,254,030	Dinactins	100%
12	Terpene	1,299,563	1,337,571	Formicamycins	18%
13	Fatty_acid	1,391,530	1,411,546	Asukamycin	9%
14	Saccharide	1,482,920	1,514,707	Dechlorocuracomycin	16%
15	RiPP	1,550,247	1,561,632	NA	
16	Saccharide	1,566,484	1,589,151	Enduracidin	14%
17	PKS	1,624,227	1,732,875	Warkmycin	97%
18	Halogenated	1,778,277	1,797,054	NA	
19	Siderophore	1,907,090	1,920,104	Ficellomycin	3%
20	Terpene	2,372,509	2,430,987	Stambomycin	16%
21	Saccharide	2,483,973	2,504,826	NA	
22	Saccharide	2,638,010	2,665,831	NA	
23	Saccharide	2,716,974	2,740,644	NA	
24	Lanthipeptide	2,771,646	2,804,777	AmfS	100%
25	Melanin	2,832,234	2,842,656	Melanin	100%
26	PKS-NRPS	2,863,825	2,952,386	Frigocyclinone	24%
27	Saccharide	3,015,862	3,043,856	Calicheamicin	2%
28	Saccharide	3,117,380	3,148,637	NA	
29	Halogenated	3,444,989	3,464,863	Caniferolide	4%
30	PKS-NRPS	3,934,309	4,114,251	RP-1776	95%
31	Ectoine	4,831,371	4,840,375	Ectoine	75%
32	PKS	5,085,935	5,173,228	Anthracimycin	73%
33	Saccharide	5,226,715	5,245,804	NA	
34	Saccharide	5,428,844	5,465,067	NA	
35	Siderophore	5,673,906	5,685,684	Desferrioxamin B	80%
36	Lanthipeptide	5,766,480	5,788,895	NA	
37	Saccharide	5,939,603	5,983,999	Phosphonoglycans	14%
38	Lanthipeptide	6,107,105	6,175,310	CDA	10%
39	Fatty_acid	6,201,945	6,221,750	NA	
40	Saccharide	6,415,616	6,444,572	Acarviostatin	33%
41	Saccharide	6,577,910	6,601,547	NA	
42	Saccharide	6,789,625	6,827,138	SF2575	6%
43	Ectoine	6,829,116	6,839,514	Ectoine	100%
44	Terpene	7,276,290	7,295,361	Steffimycin D	19%
45	NRPS	7,614,167	7,659,334	Collismycin A	14%
46	Thiopeptide	7,687,241	7,717,428	Kanamycin	8%
47	Halogenated	7,789,710	7,811,065	NA	
48	PKS	7,944,108	7,997,088	Herboxidiene	6%
49	NRPS	7,997,553	8,047,034	Retimycin A	13%
50	NRPS	8,099,304	8,191,692	Streptobactin	94%
51	Terpene	8,254,330	8,276,543	Geosmin	100%
52	Butyrolactone	8,308,010	8,318,954	Coelimycin P1	8%

### Compounds Isolated Using Genome Mining and One Strain Many Compounds Approach

**FIGURE 4:**
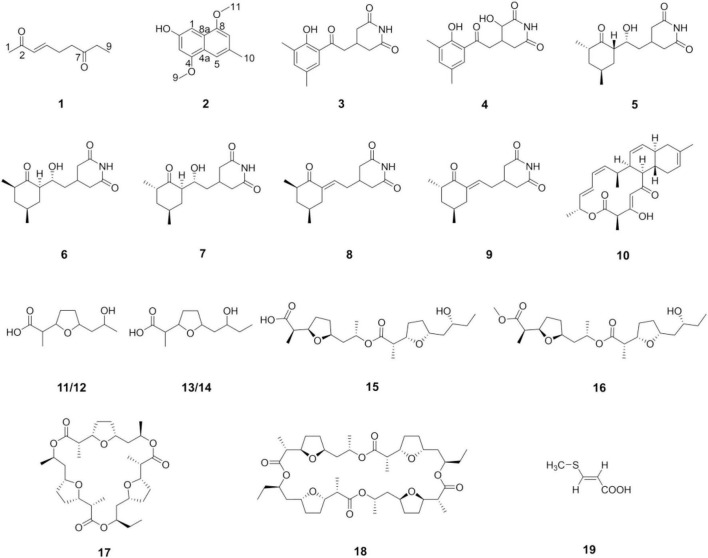
Structures of the compounds isolated from strain YINM00001.

With the guidance of the genome mining results, strain YINM00001 was fermented using OSMAC approach. Two new compounds, peperodione (**1**) and peperophthalene (**2**), together with 17 known ones, were obtained (Figure 4). Their structures were established by extensive spectroscopic analyses.

The chemical investigations of the EtOAc extract of 4# medium fermentation of strain YINM00001 were performed. This led to the discovery of a new nonane-dione, peperodione (**1**), as well as 17 known compounds, actiphenol (**3**), 3-hydroxy-3-[2-(2-hydroxy-3,5-dimethylphenyl)-2-oxoethyl] glutarimide (**4**) (Sun et al., 2014), cycloheximide (**5**) (Schaeffer and Jain, 1963), isocycloheximide (**6**) (Casey et al., 1976), naramycin B (**7**) (Johnson et al., 1965), 4-[2′-(3″(*R*),5″(*S*)-3″,5″-dimethyl-2″-oxocyclohexylidene)ethyl]piperidine-2,6-dione (**8**), 4-[2′-(3″(*S*),5″(*S*)-3″,5″-dimethyl-2″-oxocyclohexylidene)ethyl] piperidine-2,6-dione (**9**) (Guo et al., 2009), anthracimycin (**10**) (Jang et al., 2013), (±)-nonactic acid (**11/12**), (±)-homononactic acid (**13/14**) (Huang et al., 2015), feigrisolide C (**15**), methoxy-feigrisolide C (**16**) (Kim et al., 2005), bis-nonactic-homononactic trilactone (**17**) (Rezanka et al., 2004), dinactin (**18**) (Xie et al., 2014), and (*E*)-3-(methylthio)propenoic acid (**19**) (Detterbeck and Hesse, 2002). In an attempt to expand the metabolic profile, the strain was cultivated on 9# medium. Then, the research of the extract yielded a new methylnaphthalene, peperophthalene (**2**).

**FIGURE 5:**
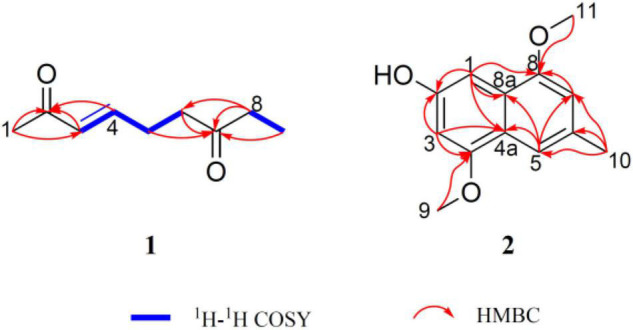
Key ^1^H-^1^H COSY and HMBC correlations of peperodione **(1)** and peperophthalene **(2)**.

Peperodione (**1**) was isolated as a colorless solid, and its molecular formula was determined to be C_9_H_14_O_2_ based on the HR-ESIMS data (*m/z* 177.0887 [M + Na]^+^, calcd for 177.0886), implying the presence of three degrees of unsaturation. The ^1^H NMR data of **1** revealed the presence of two olefin protons at δ_*H*_ 6.79 (1H, dt, *J* = 16.0, 6.4 Hz) and 6.06 (1H, d, *J* = 16.0 Hz). The ^13^C NMR data of **1** (Table 5), with the aid of DEPT and HSQC spectral analyses (Supplementary Figures 5, 7), revealed the presence of two carbonyls (at δ_*C*_ 209.8 and 198.7), two methyls (at δ_*C*_ 27.2 and 8.0), three sp^3^ methylenes (at δ_*C*_ 40.4, 36.2, and 26.4), and two sp^2^ methines (at δ_*C*_ 146.7 and 131.9). This analysis brought the total number of unsaturations to three, indicating the nature of being a chain molecule. The ^1^H-^1^H COSY spectrum (Figure 5) showed the presence of two independent spin systems, H-3/H-4/H-5/H-6 and H-8/H-9. The plane structure of **1** was confirmed by the HMBC correlations (Figure 5) from H-1 to C-2 and C-3, from H-3 and H-4 to C-2, from H-8 to C-6, as well as from H-5, H-6, H-8, and H-9 to C-7. Then, the *J*_3_,_4_ = 16.0 Hz for H-3, 4 suggested a *trans* double bond in compound **1** as depicted in Figure 4.

**TABLE 5:** ^1^H (400 MHz) and ^13^C (100 MHz) NMR data for peperodione (1) and peperophthalene (2) in CDCl_3_.

Position	1		2	
	δ_*C*_, type	δ_*H*_, (*J* in Hz)	δ_*C*_, type	δ_*H*_, (*J* in Hz)
1	27.2, CH_3_	2.23 (s)	100.7, CH	7.49 (s)
2	198.7, C		146.4 (146.44), C	
3	131.9, CH	6.06 (d, 16.0)	109.0, CH	7.14 (s)
4	146.7, CH	6.79 (dt, 16.0, 6.6)	146.4 (146.35), C	
4a			118.8, C	
5	26.4, CH_2_	2.50 (overlap)	118.3, CH	7.05 (s)
6	40.4, CH_2_	2.60 (t, 7.2)	134.3, C	
7	209.8, C		104.8, CH	6.55 (s)
8	36.2, CH_2_	2.44 (q, 7.3)	154.6, C	
8a			131.1, C	
9	8.0, CH_3_	1.07 (t, 7.3)	56.1, CH_3_	4.03 (s)
10			22.3, CH_3_	2.46 (s)
11			55.6, CH_3_	3.99 (s)

Peperophthalene (**2**) was isolated as a white solid. A molecular formula of C_13_H_14_O_3_ was assigned by interpretation of positive HRESIMS (*m/z* 219.1018 [M + H]^+^, calcd for 219.1016), indicating 7 degrees of unsaturation. The ^1^H NMR data (Table 5) displayed characteristic signals for four aromatic singlets at δ_*H*_ 7.49, 7.14, 7.05, and 6.55. The ^13^C NMR and DEPT spectral data (Table 5 and Supplementary Figure 11) allowed 13 carbon resonances to be classified into six non-protonated carbons [at δ_*C*_ 154.6 (oxygenated), 146.44 (oxygenated), 146.35 (oxygenated), 134.3, 131.1, and 118.1], four methines (at δ_*C*_ 118.3, 109.0, 104.8, and 100.7), along with three methyls [at δ_*C*_ 56.1 (oxygenated), 55.6 (oxygenated), and 22.3]. On the basis of the multiple HMBC correlations (Figure 5 and Supplementary Figure 13) from H-1 to C-2, C-4a, C-8, and C-8a, from H-3 to C-2, C-4, and C-4a, from H-5 to C-4a, C-7, and C-8a, from H-7 to C-5 and C-8, combined with from H-10 to C-5, C-6, and C-7, established the methyl naphthalene skeleton in compound **2**. Sequentially, the two methoxy groups attached at C-4 and C-9 were uncovered by the HMBC correlations from H-9 to C-4, and from H-11 to C-8, respectively (Supplementary Figure 13). The structure of **2** was therefore assigned as shown in Figure 4.

### Compounds Consisted With Gene Clusters

Several isolated compounds are consistent with highly conserved gene clusters that are identified by genome mining in the chromosome of strain YINM00001.

Cluster 2 showed 100% similarities with cycloheximide gene cluster, which was consistent with the result that strain YINM00001 could produce cycloheximide and analogs (Yin et al., 2014). These compounds belong to the glutarimide-containing polyketide family, which have been pursued to be promising anti-metastatic drug due to their potent cell migration inhibition activity and cytotoxicity. Cycloheximide could inhibit protein synthesis in eukaryote cells by efficiently inhibiting translation elongation through binding to the 60S ribosomal subunits. It was widely used as fungicide, plant growth regulator, and protein synthesis inhibitor (Schneider-Poetsch et al., 2010). The high yield of cycloheximide and analogs probably accounted for the strong inhibition of strain YINM00001 against fungal pathogens.

Cluster 11 showed 100% similarities with dinactin gene cluster, which was consistent with the result that strain YINM00001 could produce dinactin and analogs (Kwon et al., 2001). These compounds belong to type II polyketide metabolites, which had been isolated from fermentation broth of several *Streptomyces* strains. These compounds use nonactic acid and homononactic acid as building units of ionophoretic character. Dinactin and analogs exhibited a very wide range of effects, including antibacterial, antifungal, antitumor, acaricidal, insecticidal, antiprotozoan, and antiparasitic activities (Zhang et al., 2020). The presence of these dinactins coincided with the antimicrobial activity of strain YINM00001.

Cluster 32 showed 100% similarities with anthracimycin gene cluster, which was consistent with the result that strain YINM00001 could produce anthracimycin (Alt and Wilkinson, 2015). Anthracimycin and analogs, belonging to decalin-containing tricyclic macrolides, were isolated from several marine-derived actinomycete strains. Anthracimycin showed strong antibiotic activities against *Bacillus anthracis* and methicillin resistant *Staphylococcus aureus* (MRSA). Spores of *B. anthracis* have been used as bioterrorism weapons, and MRSA has become a global health challenge. Meanwhile, anthracimycin exhibits anticancer activity (Davison et al., 2020). This is the first time that it has been found from endophytic *Streptomyces* of inland plants. The gene cluster of this compound may have been passed down from ancestors to different offspring long ago, and these strains evolved independently in different environments.

Although biosynthesis of cycloheximide, dinactin, anthracimycin, and their analogs has been studied before, some important biosynthetic mechanisms remain mysterious (Kwon et al., 2001, 2002; Heine et al., 2014; Yin et al., 2014; Alt and Wilkinson, 2015). Further studies on this talented strain would help us to uncover these secrets.

## Conclusion

Endophytic bacteria were isolated from a Chinese medicinal herb *Peperomia dindygulensis* Miq. A *Streptomyces* sp. YINM00001 showed strong antimicrobial activity, and multiple antibiotic resistance was chosen for natural products exploration. After using genome mining and OSMAC approach to investigate this *Streptomyces* strain, two new compounds, peperodione (**1**) and peperophthalene (**2**), and 17 known compounds were isolated from different fermentation broth. Among them, cycloheximide, dinactin, anthracimycin, and analogs possess outstanding antimicrobial and/or anticancer activities, which had been pursued to be drug leads for a long time. The appearance of these compounds is not surprising due to the identification of their biosynthetic gene clusters through genome mining before fermentation. The large amount and high diversity of cycloheximide, dinactin, anthracimycin, and analogs produced by strain YINM00001 highlight this talented strain as an ideal candidate for further biosynthetic studies and production improvement of these valuable compounds. Other gene clusters might be silent under proceeding fermentation conditions. Further studies, such as heterologous expression and genetic modification, are needed to explore more novel compounds. Natural products from *Peperomia dindygulensis* endophytic bacteria, especially strain YINM00001, might provide a partial function of this medicinal herb to cure diseases, including cough, asthma, measles, burns, and cancers.

In conclusion, new drug leads research needs to be accelerated to counter the threat of dangerous infectious diseases and cancer. Endophytic *Streptomyces* sp. YINM00001 is a promising candidate to discover valuable secondary metabolites. Using genome mining and OSMAC approach together to investigate new strains from new habitats is a promising way to explore novel natural products.

## Data Availability Statement

The complete chromosome and plasmid sequences of strain YINM00001 were deposited in GenBank under accession number CP086102.1 and CP086103.1. This strain had been deposited at the Yunnan University and Guangdong Microbial Culture Collection Center under accession number YINM00001 and GDMCC No. 61693, respectively. The datasets presented in this study can be found in online repositories. The names of the repository/repositories and accession number(s) can be found in the article/Supplementary Material.

## Author Contributions

MY, HZ, and Z-TD designed the study, carried out the data analysis, and wrote the manuscript. TL, ZR, W-XC, G-DL, Z-T-LZ, H-BS, MeiW, T-PX, MengW, and J-YC carried out the experiments and participated in data analysis. All authors have read and approved the manuscript.

## Conflict of Interest

The authors declare that the research was conducted in the absence of any commercial or financial relationships that could be construed as a potential conflict of interest.

## Publisher’s Note

All claims expressed in this article are solely those of the authors and do not necessarily represent those of their affiliated organizations, or those of the publisher, the editors and the reviewers. Any product that may be evaluated in this article, or claim that may be made by its manufacturer, is not guaranteed or endorsed by the publisher.

## Footnotes

1
https://github.com/tseemann/barrnap

2
http://www.repeatmasker.org
