# Supplementary material for: Exploration of Diverse Secondary Metabolites From Streptomyces sp. YINM00001, Using Genome Mining and One Strain Many Compounds Approach
Source: Front Microbiol. 2022 Feb 10;13:831174. doi: 10.3389/fmicb.2022.831174 (PMC8866825; doi:10.3389/fmicb.2022.831174)
Supplement: Supplementary file 1 [file Data_Sheet_1.docx]

Supplementary Material

**Exploration of Diverse Secondary Metabolites from *Streptomyces* sp. YINM00001, Using Genome Mining and OSMAC Approach**

Tao Liu ^a, 1^, Zhen Ren^b, 1^，Wei-Xun Chunyu^c, 1^, Gui-Ding Li^a^, Xiu Chen^b^, Zhou-Tian-Le Zhang^a^, Hui-Bing Sun^a^, Mei Wang^a^, Tian-Peng Xie^a^, Meng Wang^a^, Jing-Yuan Chen^a^, Hao Zhou^a, *^ and Zhong-Tao Ding ^a, d *^ Min Yin ^a, *^

^a^ School of Medicine, School of Chemical Science and Technology, Yunnan University, 2 North Cui Hu Road, Kunming, Yunnan 650091, China

^b^ School of Agriculture and Life Sciences, Kunming University, 2 Pu Xin Road, Kunming, Yunnan 650214, China

^c^ Department of Pathogen Biology and Immunology, Kunming Medical University, Kunming, Yunnan 650500, China

^d^ College of Pharmacy, Dali University, Dali, Yunnan 671000, China

*Corresponding authors. E-mail address: haozhou@ynu.edu.cn (H. Zhou), ztding@ynu.edu.cn (Z. Ding) and yinmin@ynu.edu.cn (M. Yin); mailing address: Yunnan University, 2 North Cui Hu Road, Kunming, Yunnan 650091, China

^1^ These authors contributed equally to this study


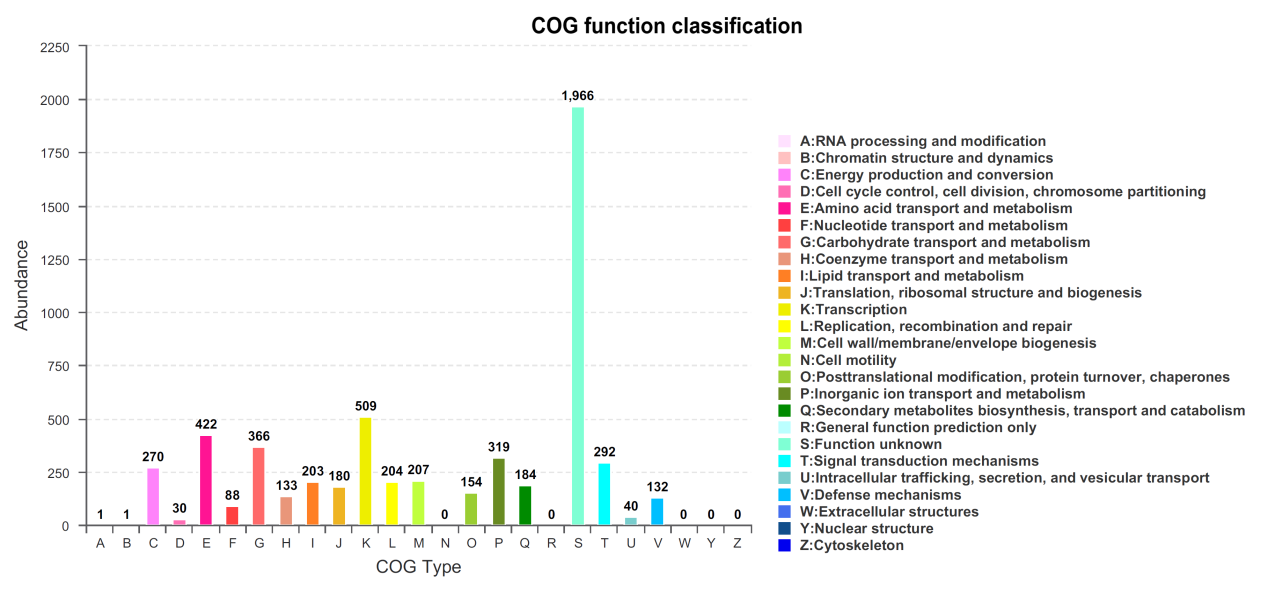


**Figure S1** COG-based functional classification of genes located on YINM00001 chromosome.


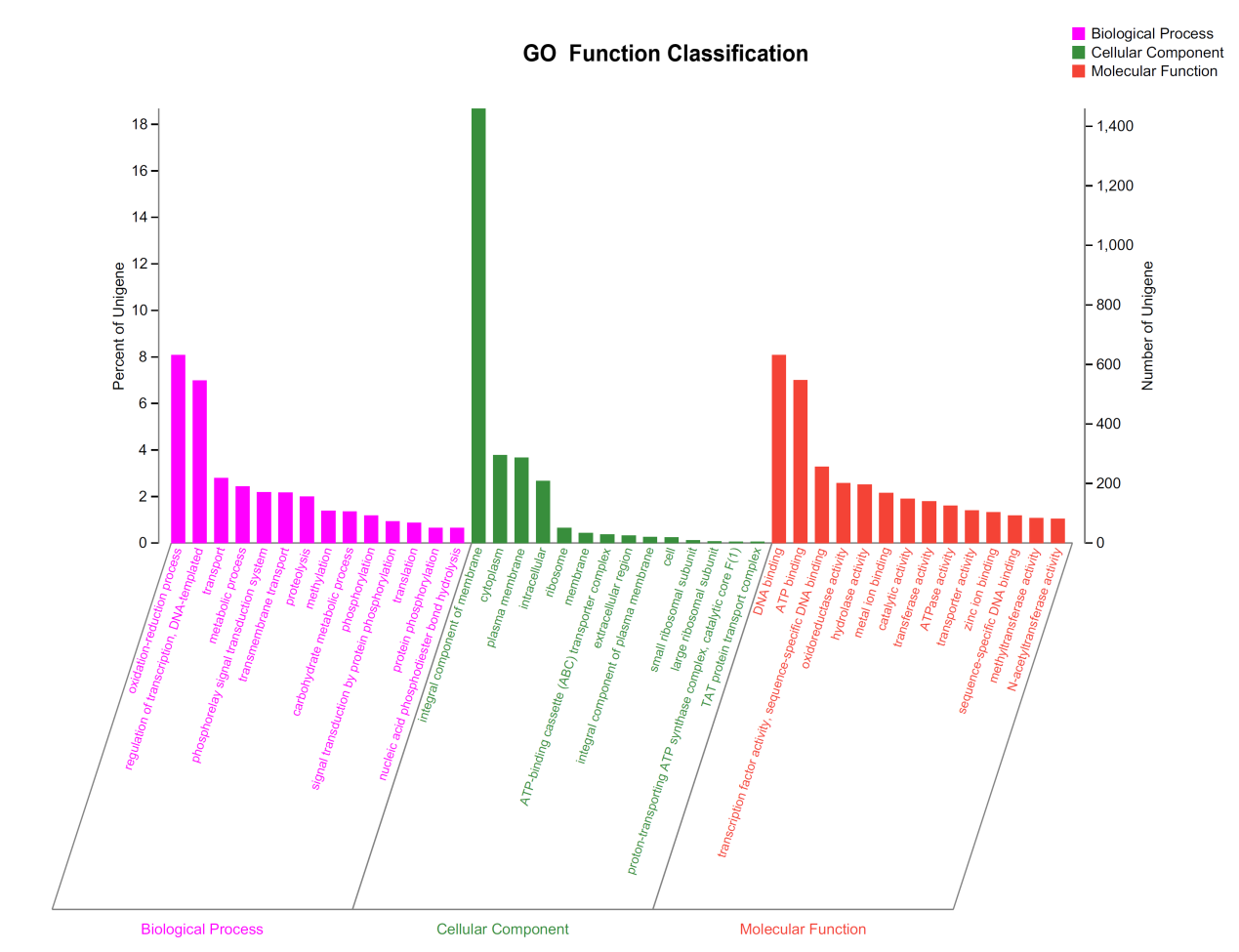


**Figure S2** GO-based functional classification of genes located on YINM00001 chromosome.


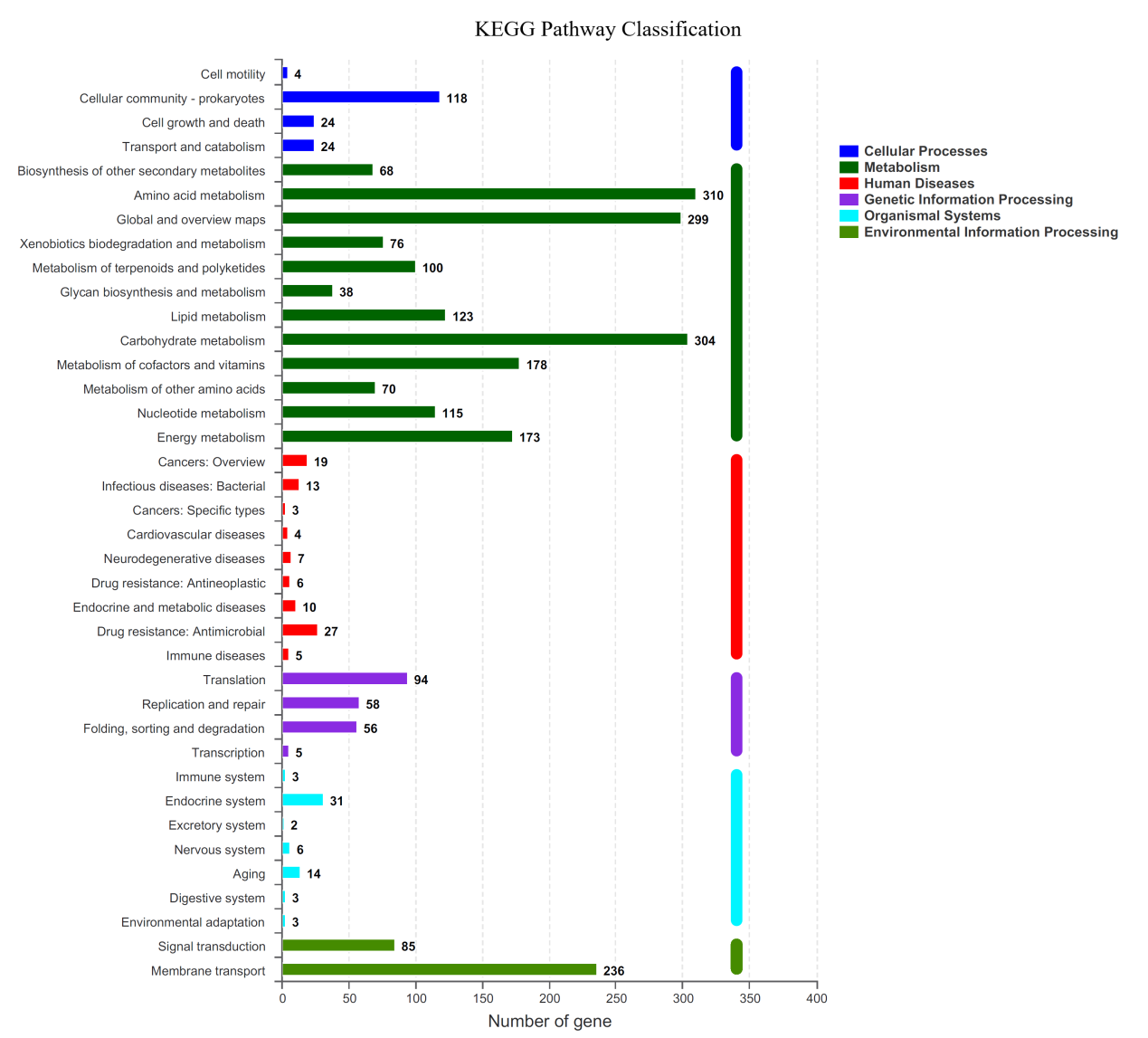


**Figure S3** KEGG-based functional classification of genes located on YINM00001 chromosome.

**Figure S4** ^1^H-NMR spectrum of peperodione (**1**) in CDCl_3_ (400 MHz).

**Figure S5** ^13^C-NMR and DEPT spectra of peperodione (**1**) in CDCl_3_ (100 MHz).

**Figure S6** ^1^H-^1^H COSY spectrum of peperodione (**1**) in CDCl_3_ (400 MHz).

**Figure S7** HSQC spectrum of peperodione (**1**) in CDCl_3_ (^1^H-400 MHz).

**Figure S8** HMBC spectrum of peperodione (**1**) in CDCl_3_ (^1^H-400 MHz).

**Figure S9** (+)-HRESIMS data of peperodione (**1**).

**Figure S10** ^1^H-NMR spectrum of peperophthalene (**2**) in CDCl_3_ (400 MHz).


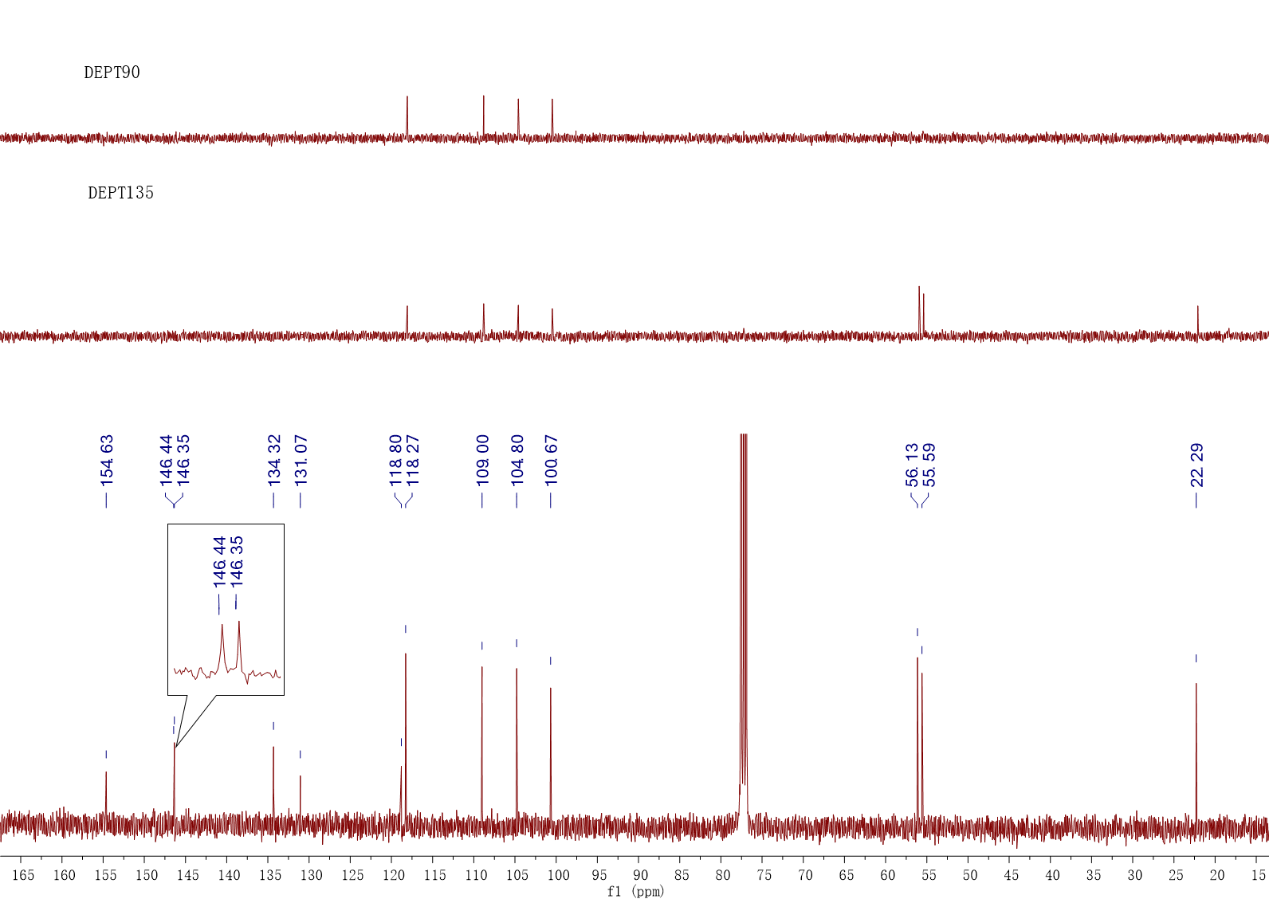


**Figure S11** ^13^C-NMR and DEPT spectra of peperophthalene (**2**) in CDCl_3_ (100 MHz).

**Figure S12** HSQC spectrum of peperophthalene (**2**) in CDCl_3_ (^1^H-400 MHz).


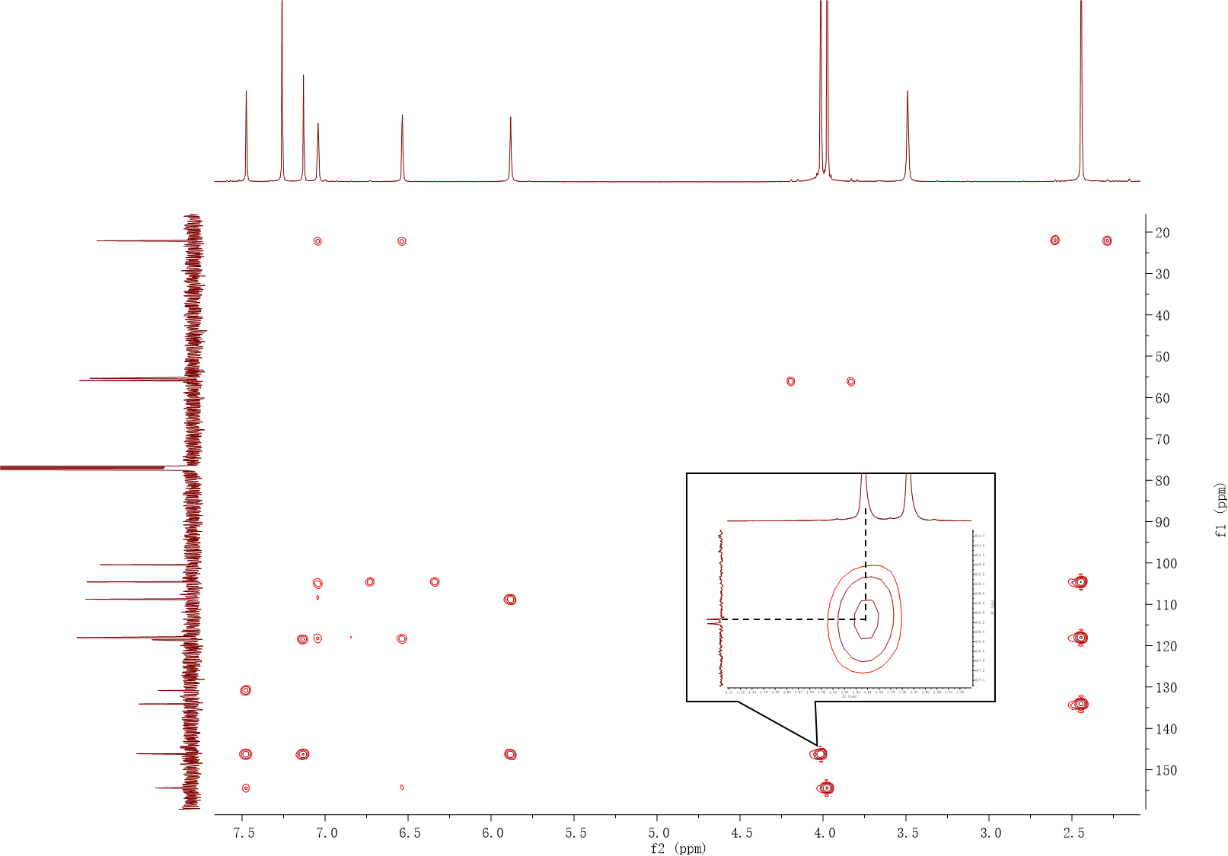


**Figure S13** HMBC spectrum of peperophthalene (**2**) in CDCl_3_ (^1^H-400 MHz).

**_
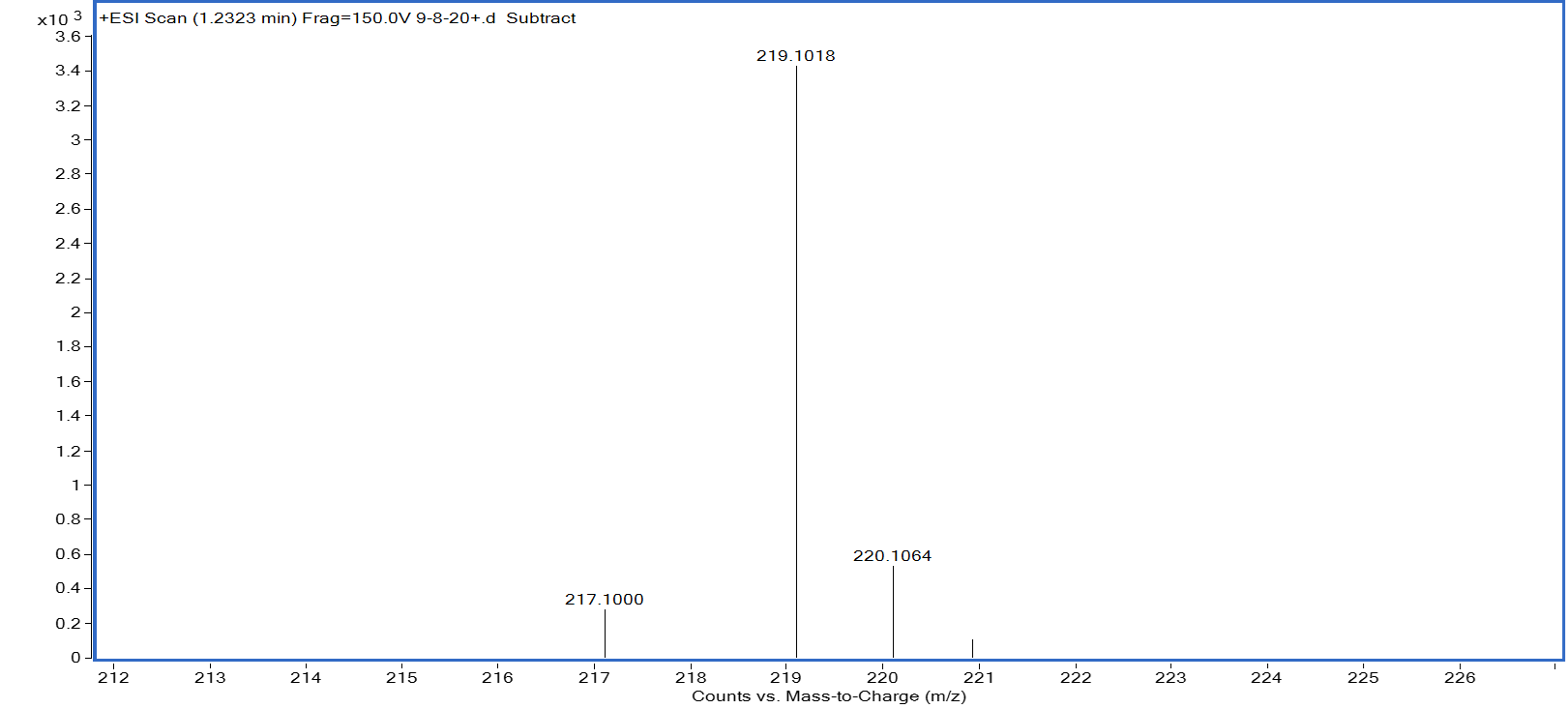
_**

**Figure S14** (+)-HRESIMS data of peperophthalene (**2**).
